# AMH independently predicts aneuploidy but not live birth per transfer in IVF PGT-A cycles

**DOI:** 10.1186/s12958-023-01066-w

**Published:** 2023-02-04

**Authors:** Howard J. Li, David B. Seifer, Reshef Tal

**Affiliations:** Dept. of Obstetrics, Gynecology & Reproductive Sciences, Yale School of Medicine, 333 Cedar Street, New Haven, CT 06510 U.S.A.

**Keywords:** Anti-Mullerian hormone (AMH), Pre-implantation genetic testing for aneuploidy (PGT-a), Pre-implantation genetic diagnosis (PGD), Aneuploidy, In-vitro fertilization, Live birth

## Abstract

**Background:**

While anti-Müllerian hormone (AMH) predicts quantitative IVF outcomes such as oocyte yield, it is not certain whether AMH predicts markers of oocyte quality such as aneuploidy.

**Methods:**

Retrospective case–control analysis of the SART-CORS database, 2014–2016, to determine whether anti-Müllerian hormone (AMH) predicts aneuploidy and live birth in IVF cycles utilizing preimplantation genetic testing for aneuploidy (PGT-A).

**Results:**

Of 51,273 cycles utilizing PGT-A for all embryos, 10,878 cycles were included in the final analysis; of these, 2,100 cycles resulted in canceled transfer due to lack of normal embryos and 8,778 cycles resulted in primary FET. AMH levels of cycles with ≥ 1 euploid embryo were greater than those of cycles with no normal embryos, stratifying by number of embryos biopsied (1–2, 3–4, 5–6, and ≥ 7), *P* < 0.017 for each stratum. Adjusting for age and number of embryos biopsied, AMH was a significant independent predictor of ≥ 1 euploid embryo for all age groups: < 35 yrs (aOR 1.074; 95%CI 1.005–1.163), 35–37 years (aOR 1.085; 95%CI 1.018–1.165) and ≥ 38 years (aOR 1.055; 95%CI 1.020–1.093). In comparative model analysis, AMH was superior to age as a predictor of  ≥ 1 euploid embryo for age groups < 35 years and 35–37 years, but not  ≥ 38 years. Across all cycles, age (aOR 0.945, 95% CI 0.935–0.956) and number of embryos (aOR 1.144, 95%CI 1.127–1.162) were associated with live birth per transfer, but AMH was not (aOR 0.995, 95%CI 0.983–1.008). In the subset of cycles resulting in ≥ 1 euploid embryo for transfer, neither age nor AMH were associated with live birth.

**Conclusions:**

Adjusting for age and number of embryos biopsied, AMH independently predicted likelihood of obtaining ≥ 1 euploid embryo for transfer in IVF PGT-A cycles. However, neither age nor AMH were predictive of live birth once a euploid embryo was identified by PGT-A for transfer. This analysis suggests a predictive role of AMH for oocyte quality (aneuploidy risk), but not live birth per transfer once a euploid embryo is identified following PGT-A.

## Background

Anti-Müllerian hormone (AMH) is a well-established marker for ovarian reserve and found to correlate with several outcomes in reproductive medicine, most reliably oocyte yield during ART cycles [1–4]. AMH, by some studies, has been shown to have a moderate association with implantation, clinical pregnancy, and live birth for both fresh and frozen transfers [5–11]. It has also been shown to correlate with amenorrhea and PCOS severity, risk of premature ovarian insufficiency, and onset of menopause [12–14]. For its ease of use, low menstrual variability, and high predictive value for IVF outcomes, AMH has become the most widely used marker of ovarian reserve particularly in the IVF setting, supplanting other markers of ovarian reserve such as antral follicle count (AFC), clomiphene citrate testing, day 3 FSH, and inhibin B [4, 15].

While the relationship between AMH and quantitative IVF outcomes such as oocyte and embryo yield is well-established and reliably reproducible across multiple studies [15, 16], whether AMH is also predictive of oocyte quality remains unclear. It is known that advancing age is associated with a decline in ovarian reserve markers (including AMH), oocyte quantity, and oocyte quality [17, 18]. Whether these trends are biologically-coupled or independent processes confounded by age is not well understood, and studies attempting to answer the “quality vs. quantity” question thus far have had mixed results [15].

AMH has been shown to predict oocyte morphology, fertilization, blastocyst formation, implantation, and pregnancy rates [5, 19, 20]. Borges et al. (2017) found that, in a sample of 4488 oocytes from 408 patients, AMH was predictive of embryo morphology on day 2 and day 3, fertilization and blastocyst formation, implantation rates, and clinical pregnancy rates after adjusting for age [5]. At the same time, other reports have found no or partial associations between ovarian reserve and markers of embryo quality [21–25]. Morin et al. (2018) found among 2,103 patients undergoing IVF, adjusted odds of blastocyst development, aneuploidy, and live birth after transfer were similar between patients with AMH levels under the 10^th^ percentile and patients with AMH between the 25-75^th^ percentiles [22].

In the last two years, recent studies have also noted negative or mixed results. A 2022 study of 231 patients found only an association between AMH and Day 5 embryo quality, but not between AMH and Day 3 embryo quality or clinical pregnancy rate [26]. Another study found that, among 521 patients, AMH was predictive of oocytes retrieved, but not predictive of obtaining “good quality” embryos [27]. In an analysis of 492 IVF/ICSI cycles, patients with low AMH (< 1.1 ng/ml) had greater rates of cycle cancelation, fewer oocytes, and slightly lower rates of Grade I-II embryo formation, but no differences in fertilization, blastocyst formation, implantation rates, as well rates of miscarriage and livebirth.

The wide heterogeneity of these study results may be due in part to varying criteria used for diagnosing DOR and multiple metrics for assessing oocyte quality. Variables most intrinsic to oocyte quality (fertilization and blastocyst formation, oocyte and embryo morphology) may be far removed from more clinically meaningful outcomes such as live birth. Indeed, studies that find significant associations between DOR and morphologic makers have commonly found no effect on subsequent pregnancy and live birth rates. At the same time, other studies have reported associations between DOR and clinical pregnancy, miscarriage, and live birth rates (both per cycle and cumulative), but the impact of oocyte quality on these multifactorial outcomes are often intertwined with effects extrinsic to the individual oocyte (oocyte yield, maternal factors).

Embryo aneuploidy is an objective qualitative factor that is directly affected by intrinsic oocyte quality with definitive implications for ultimate IVF outcome, and now increasingly assessed via the use of preimplantation genetic testing for aneuploidy (PGT-A). Although its use and clinical utility remain controversial [28–30], the differentiation between PGT and non-PGT cycles in the SART-CORS data base beginning in 2014 provides an opportunity to correlate AMH and risk of aneuploidy as assessed by PGT.

Katz-Jaffe et al. (2013) found in a prospective cohort of 372 patients that patients with AMH < 1 ng/ml had a higher percentage of aneuploid embryos as assessed by PGT, though these effects were not necessarily independent of age [31]. Jaswa et al. (2021) found that among 1152 women undergoing IVF, women with DOR had 24% reduced odds of a single biopsied blastocyst being euploid after adjusting for age [32]. Results are highly suggestive that, independent of age, DOR is associated with increased risk of aneuploid embryos. However, in using a comprehensive definition of DOR via the Bologna criteria, the predictive value of AMH alone was not reported in this study.

With the important exception of two recent smaller studies—one comparing women < 38 years with low and normal AMH [22], and another comparing women ≤ 40 years with and without physician-reported DOR or poor ovarian response [33]—that reported no association between DOR and aneuploidy rates as determined by PGT, the evidence thus far suggests that, at least with the specific concern of embryo ploidy, DOR appears to be associated with increased risk of aneuploidy. However, larger scale studies are needed to further investigate the association between aneuploidy risk and DOR, especially as determined by AMH, now the most contemporary clinical marker of DOR. Such findings would have important implications for the counseling and management of patients with DOR, particularly in identifying which patients would benefit from PGT-A.

## Methods

Data from the Society for Assisted Reproductive Technology Clinic Outcome Reporting System (SART-CORS) database, 2014–2016, were analyzed. Data were collected through voluntary submission, verified by SART, and reported to the Centers for Disease Control and Prevention (CDC) in compliance with the Fertility Clinic Success Rate and Certification Act of 1992 (Public Law 102-493). SART maintains HIPAA-compliant business associates agreements with reporting clinics. In 2004, following a contract change with the CDC, SART gained access to the SART CORS data system for the purposes of conducting research. SART requests the following information be provided in all studies using the analysis and publication of SARTCORS data. During 2019, 81% of clinics were SART members reporting 90% of all IVF cycles in the United States. The data in the SART CORS are validated annually with some clinics receiving on-site visits for chart review based on an algorithm for clinic selection. During each visit, data reported by the clinic were compared with information recorded in patients’ charts. In 2021, records for 1,945 cycles at 33 clinics were randomly selected for full validation, along with 262 fertility preservation cycles selected for partial validation. Nine out of ten data fields selected for validation were found to have discrepancy rates of ≤ 5%. The exception was the diagnosis field, which, depending on the diagnosis, had a discrepancy rate between 0.7% and 9.1%.

Cycles were included in the analysis if PGT was performed on all embryos, and only cycles utilizing PGT-A were included. Cycles utilizing other PGT applications (PGT-M, PGT-HLA) were excluded. Of these, cycles resulting in the following outcomes were selected for a case–control study design: 1. No transfer attempted due to no normal embryos after PGT-A, or 2. Transfer attempted (of a presumed euploid embryo) following PGT-A of all embryos. Cycles were only included in the final analysis if the number of embryos biopsied was recorded, and if an AMH value was available within 1 year of the index oocyte retrieval.

Demographic variables (age, race), body mass index (BMI), AMH, etiology of infertility, and stimulation characteristics (FSH dosage, number of oocytes retrieved, embryos cryopreserved) are reported with summary statistics.

Distribution of AMH values were compared using Mann–Whitney testing between cycles resulting in an attempted transfer and cycles resulting in an aborted transfer. Analysis was stratified by number of embryos biopsied (1–2, 3–4, 5–6, and ≥ 7 embryos) and by age (< 35, 35–37, ≥ 38 years at time of index cycle start). Likelihood of ≥ 1 euploid embryo for transfer and live birth following PGT-A were modeled with multivariable logistic regression using age, AMH, and number of embryos biopsied as independent variables. Separate models were also fitted for each of 3 age strata: < 35, 35–37, ≥ 38 years at time of cycle start. Comparative model analysis was performed using Likelihood Ratio (LR) testing for nested models, and Akaike information criterion (AIC) for non-nested models.

Data are expressed as mean with standard deviation (SD) or median with interquartile range (IQR). Differences between groups in continuous variables are compared using Student’s T or Mann–Whitney testing. Differences in categorical variables are compared using Chi-squared testing. *P*-values < 0.05 were considered statistically significant. Statistical analyses were performed in GraphPad Prism version 9.4.1 (La Jolla, CA) and R v3.4.1 (Vienna, Austria).

## Results

### Cycles included for analysis

**Fig. 1:**
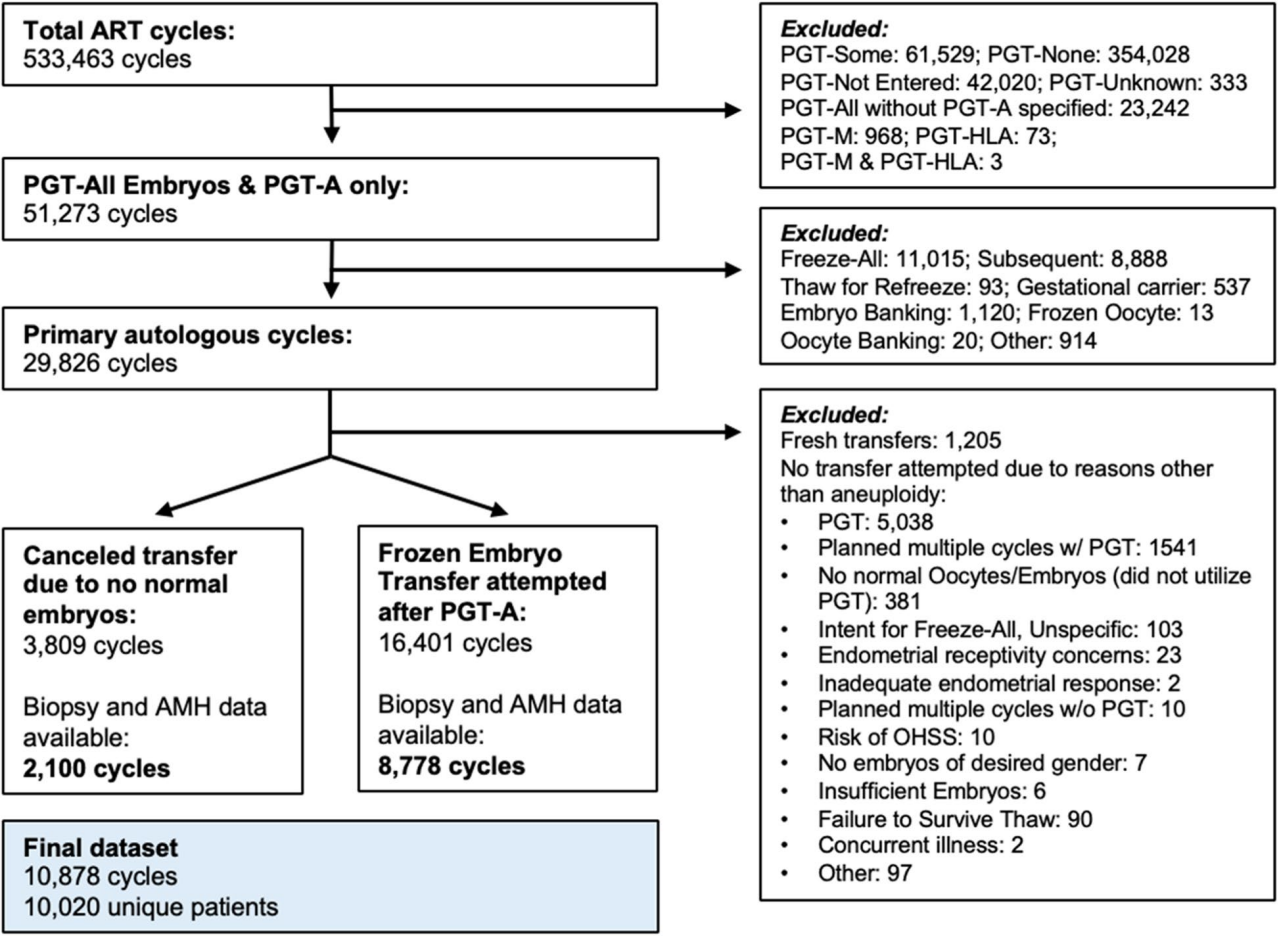
Flowchart of included cycles

Of 533,463 IVF cycles in the SART-CORS database (2014–2016), there were 29,826 primary autologous cycles utilizing PGT-A for all embryos. Of these, 3,809 cycles resulted in a canceled transfer due no normal embryos after PGT-A and 16,401 cycles resulted in an attempted transfer of a presumed euploid embryo after PGT-A. After excluding cycles without the number of embryos biopsied documented or without an AMH value within 1 year of index cycle start, 10,878 cycles representing 10,020 unique patients were included in the final analysis: 2,100 cycles with aborted transfers due to no normal embryos after PGT-A, and 8,778 cycles with an attempted transfer after PGT-A. There were 4,893 live births (55.8% of transfer cycles). Flowchart of included and excluded cycles is show in Fig. 1. Baseline cycle characteristics are summarized in Table 1.

**Table 1:** Baseline and stimulation cycle characteristics

	No normal embryos (*n* = 2100)	≥ 1 presumed euploid embryo (*n* = 8778)	
Age (years)	40.0 (3.2)	37.0 (3.7)	*P* < 0.001
AMH (ng/ml)	1.9 (2.4)	3.5 (3.5)	*P* < 0.001
BMI (kg/m^2^)	24.9 (5.2)	24.9 (5.3)	*P* = 0.58
Race			*P* < 0.001
*White*	798 (38.0%)	3382 (38.5%)	
*American Indian/Alaskan Native*	1 (0.05%)	8 (0.1%)	
*Asian*	266 (12.7%)	938 (10.7%)	
*Black/ African American*	84 (4.0%)	246 (2.8%)	
*Hispanic/Latina*	99 (4.7%)	331 (3.8%)	
*Hawaiian/ Pacific Islander*	4 (0.2%)	9 (0.1%)	
*Multiracial*	11 (0.5%)	49 (0.6%)	
*Unknown*	837 (39.9%)	3815 (43.5%)	
Infertility etiology			
*Male factor*	617 (29.4%)	2640 (30.1%)	*P* = 0.49
*Endometriosis*	139 (6.6%)	596 (6.8%)	*P* = 0.82
*PCOS*	81 (3.9%)	945 (10.8%)	*P* < 0.001
*DOR*	1289 (61.4%)	2908 (33.1%)	*P* < 0.001
*Tubal factor*	200 (9.5%)	859 (9.8%)	*P* = 0.75
*Uterine factor*	283 (13.5%)	833 (9.5%)	*P* < 0.001
*Unexplained*	116 (5.5%)	978 (11.1%)	*P* < 0.001
Stimulation characteristics			
FSH dosage (IU)	4406 (1549)	3669 (1577)	*P* < 0.001
No. eggs retrieved	10.1 (6.7)	16.4 (9.0)	*P* < 0.001
No. embryos cryopreserved	2.5 (1.7)	4.6 (2.4)	*P* < 0.001

### Cycles resulting in no euploid embryos are associated with lower AMH values

Stratifying by number of embryos biopsied (1–2, 3–4, 5–6, ≥ 7), AMH levels of cycles with ≥ 1 euploid embryo for attempted transfer were greater than those of cycles with no normal embryos (Table 2a), *P* < 0.002 for each stratum of embryos biopsied. Further stratification by age (< 35, 35–37, ≥ 38 years) was also performed with analogous results, though differences in AMH between cycles resulting in canceled transfer due to no euploid embryos vs. ≥ 1 euploid embryo for attempted transfer were no longer significant for select strata due to diminished cell size (Table 2b-d).

**Table 2:** Median AMH of cycles resulting in no normal embryos vs. ≥ 1 euploid embryo after PGT-A

Embryos	No normal embryos (n)	≥ 1 euploid embryo (n)	Median AMH (No normal embryos)	Median AMH (≥ 1 euploid embryo)	*P*-value
**a. All cycles**					
1—2	1321	1919	1.2 (0.6—2.0)	1.5 (0.8—2.7)	5.86E-13
3—4	522	2463	1.6 (0.9—2.8)	2.1 (1.2—3.5)	1.02E-10
5—6	175	1848	2.3 (1.2—3.6)	2.7 (1.6—4.3)	1.71E-03
7 +	82	2548	2.9 (1.6—5.0)	4.0 (2.6—6.2)	7.71E-05
**b. Age < 35**					
1—2	63	287	1.6 (0.8—2.7)	2.0 (1.0—3.7)	0.03
3—4	32	461	2.0 (1.3—3.3)	2.9 (1.6—5.2)	0.02
5—6	15	441	4.0 (2.5—6.0)	3.2 (1.9—5.4)	0.32
7 +	7	796	5.1 (3.3—7.5)	4.5 (3.1—7.3)	0.86
**c. Age 35–37**					
1—2	162	480	1.2 (0.6—2.3)	1.6 (0.8—2.8)	5.34E-03
3—4	66	682	1.6 (1.1—2.5)	2.2 (1.3—3.7)	6.62E-03
5—6	14	522	2.2 (1.2—3.9)	2.7 (1.7—4.4)	0.384
7 +	13	735	1.8 (1.2—2.8)	4.0 (2.5—6.2)	1.36E-04
**d. Age** ≥ **38**					
1—2	1096	1152	1.1 (0.6—2.0)	1.4 (0.7—2.3)	1.28E-05
3—4	424	1320	1.6 (0.9—2.8)	1.8 (1.1—2.9)	1.42E-03
5—6	146	885	2.2 (1.1—3.5)	2.5 (1.5—3.9)	0.014
7 +	62	1017	3.0 (1.7—5.1)	3.7 (2.3—5.6)	0.054

### Multivariable logistic regression of AMH and likelihood of obtaining ≥ 1 euploid embryo for transfer

Multivariable logistic models for likelihood of ≥ 1 euploid embryo were fitted for each of 3 age groups: < 35, 35–37, and ≥ 38 years (Table 3). Adjusting for age and number of embryos biopsied, AMH was a significant independent predictor of ≥ 1 euploid embryo for all age groups: < 35 yrs (aOR 1.074; 95%CI 1.005–1.163), 35–37 years (aOR 1.085; 95%CI 1.018–1.165) and ≥ 38 years (aOR 1.055; 95%CI 1.020–1.093). Age was a predictor of ≥ 1 euploid embryo for 35–37 years (aOR 0.813; 95% CI 0.679–0.969) and ≥ 38 years (aOR 0.710; 95% CI 0.685–0.736), but not for < 35 years (aOR 1.040; 95% CI 0.953–1.130).

**Table 3:** Multivariable logistical regression

				Model comparison (LR test)		Model comparison (AICc test)	
				Model 1: Age, AMH, Embryos Model 2: Age, Embryos		Model 1: AMH, Embryos Model 2: Age, Embryos	
**Age < 35**							
	Variable	Estimate	95% CI	Model 1 ROC AUC	0.806	Model 1 ROC AUC	0.805
β1	Age	1.040	0.9526 to 1.130	Model 2 ROC AUC	0.800	Model 2 ROC AUC	0.800
β2	AMH	1.074	1.005 to 1.163	Preferred model	Model 1	Preferred model	Model 1
β3	Embryos	1.607	1.449 to 1.799	Likelihood ratio	4.495	Model 1 probability	86.42%
				*P*-value	*P* = 0.0340	Model 2 probability	13.58%
						ΔAICc	-3.702
**Age 35–37**							
	Variable	Estimate	95% CI	Model 1 ROC AUC	0.807	Model 1 ROC AUC	0.807
β1	Age	0.813	0.679 to 0.969	Model 2 ROC AUC	0.805	Model 2 ROC AUC	0.805
β2	AMH	1.085	1.018 to 1.165	Preferred model	Model 1	Preferred model	Model 1
β3	Embryos	1.729	1.584 to 1.897	Likelihood ratio	6.671	Model 1 probability	66.24%
				*P*-value	*P* = 0.0098	Model 2 probability	33.76%
						ΔAICc	-1.348
**Age ≥ 38**							
	Variable	Estimate	95% CI	Model 1 ROC AUC	0.791	Model 1 ROC AUC	0.754
β1	Age	0.710	0.686 to 0.736	Model 2 ROC AUC	0.790	Model 2 ROC AUC	0.790
β2	AMH	1.055	1.020 to 1.093	Preferred model	Model 1	Preferred model	Model 2
β3	Embryos	1.500	1.447 to 1.558	Likelihood ratio	10	Model 1 probability	< 0.01%
				*P*-value	*P* = 0.0016	Model 2 probability	> 99.99%
						ΔAICc	382.1
**All cycles**							
	Variable	Estimate	95% CI	Model 1 ROC AUC	0.829	Model 1 ROC AUC	0.789
β1	Age	0.790	0.774 to 0.805	Model 2 ROC AUC	0.828	Model 2 ROC AUC	0.828
β2	AMH	1.056	1.027 to 1.087	Preferred model	Model 1	Preferred model	Model 2
β3	Embryos	1.543	1.495 to 1.595	Likelihood ratio	15.97	Model 1 probability	< 0.01%
				*P*-value	*P* < 0.0001	Model 2 probability	> 99.99%
						ΔAICc	691.7

### Comparative Model Analysis: Age and AMH as predictors of aneuploidy risk

To compare the incremental predictive value of AMH for aneuploidy risk, a multivariable logistic model of likelihood of ≥ 1 euploid embryo fitted using age, number of embryos biopsied, and AMH was compared with a model incorporating only age and number of embryos biopsied (without AMH). In Likelihood Ratio (LR) testing, the addition of AMH significantly improved model performance for all age groups: age < 35 years (*P* = 0.034, LR 4.495, AUC_1_ = 0.806, AUC_2_ = 0.800), 35–37 years (*P* = 0.010, LR 6.671, AUC_1_ = 0.807, AUC_2_ = 0.805), and ≥ 38 years (*P* = 0.002, LR 10, AUC_1_ = 0.791, AUC_2_ = 0.790), and all cycles combined (*P* < 0.0001, LR 15.97, AUC_1_ = 0.829, AUC_2_ = 0.828).

To compare the relative predictive values of age and AMH for aneuploidy risk, a multivariable logistical model of likelihood of ≥ 1 euploid embryo fitted using number of embryos biopsied and AMH (but not age) was compared with a model incorporating number of embryos biopsied and age (but not AMH) using Akaike information criterion (AIC). Across all cycles, age was superior to AMH as a predictor of ≥ 1 euploid embryo (ΔAICc =  + 691.7, AUC_1_ = 0.789, AUC_2_ = 0.828). Stratifying by age, AMH was superior to age for age groups < 35 years (ΔAICc = -3.70, AUC_1_ = 0.805, AUC_2_ = 0.800) and 35–37 years (ΔAICc = -1.35, AUC_1_ = 0.807, AUC_2_ = 0.805) but age was superior to AMH in the subset of women ≥ 38 years (ΔAICc =  + 382.1, AUC_1_ = 0.754, AUC_2_ = 0.790).

### Multiple logistic regression of AMH and live birth

Across all cycles, age (aOR 0.945, 95%CI 0.935–0.956) and number of embryos (aOR 1.144, 95%CI 1.127–1.162) were associated with live birth per transfer, but not AMH (aOR 0.995, 95%CI 0.983–1.008) (Table 4). In the subset of cycles resulting in ≥ 1 euploid embryo for transfer, neither age (aOR 0.994, 95%CI 0.983–1.006) nor AMH (aOR 1.006, 95%CI 0.994–1.019) were associated with live birth. In this subset, the fitted model incorporating age and AMH as predictors was not predictive of live birth (AUC 0.515, 95%CI 0.503–0.527).

**Table 4:** AMH and Live Birth

				Model comparison (LR test)		Model comparison (AICc test)	
**All cycles**				Model 1: Age, AMH, Embryos Model 2: Age, Embryos		Model 1: AMH, Embryos Model 2: Age, Embryos	
	Variable	Estimate	95% CI	Model 1 ROC AUC	0.641	Model 1 ROC AUC	0.632
β1	Age	0.945	0.935 to 0.956	Model 2 ROC AUC	0.642	Model 2 ROC AUC	0.642
β2	AMH	0.995	0.983 to 1.008	Preferred model	Model 2	Preferred model	Model 2
β3	Embryos	1.144	1.127 to 1.162	Likelihood ratio	0.5256	Model 1 probability	< 0.01%
				*P*-value	*P* = 0.469	Model 2 probability	> 99.99%
						ΔAICc	103.9
				Model comparison (LR test)		Model comparison (AICc test)	
**Given ≥ 1 euploid embryo**				Model 1: Age, AMH Model 2: Age		Model 1: AMH Model 2: Age	
	Variable	Estimate	95% CI	Model 1 ROC AUC	0.515	Model 1 ROC AUC	0.512
β1	Age	0.994	0.983 to 1.006	Model 2 ROC AUC	0.509	Model 2 ROC AUC	0.509
β2	AMH	1.006	0.994 to 1.019	Preferred model	Model 2	Preferred model	Model 2
				Likelihood ratio	0.8664	Model 1 probability	49.47%
				*P*-value	*P* = 0.352	Model 2 probability	50.53%
						ΔAICc	0.0427

## Discussion

Using a large, national, standardized, multicenter database (SART-CORS), these data demonstrate that AMH predicts the likelihood of obtaining ≥ 1 euploid embryo in IVF PGT-A cycles independent of age and number of embryos biopsied. When directly comparing the predictive values of age or AMH, AMH was a superior predictor of aneuploidy for patients < 38 years, with age being far more predictive of aneuploidy risk in patients ≥ 38 years. Across all cycles, age, but not AMH, was predictive of live birth; however, in the subset of cycles for which ≥ 1 euploid embryo was obtained for transfer, neither age nor AMH predicted live birth. In other words, once a euploid embryo was identified by PGT-A, its chance of successful implantation and progression to live birth was independent of age or AMH.

This study’s greatest strength is its large sample size. With 10,778 included cycles, the sample size of this study is 1–2 orders of magnitude greater than other recent studies investigating the relationship between diminished ovarian reserve and oocyte quality including embryo aneuploidy [22–27, 31, 32]. Several limitations are acknowledged. While our primary concern was to investigate the association of AMH and oocyte quality using embryo aneuploidy as a proxy, the genetic results of individual embryos were not available, and the dichotomous outcomes of obtaining no normal embryos following PGT-A versus obtaining ≥ 1 presumed euploid embryo for transfer, controlling for the number of embryos biopsied, was used as an imperfect proxy of aneuploidy risk. Specifically, the outcome of “no normal embryos following PGT” does not sufficiently discriminate between true euploid, aneuploid, and mosaic results for which the decision to proceed with embryo transfer may vary between patients and institutions [34, 35]. However, we assume that the vast majority of PGT-tested embryo transfers in the 2014–2016 SART dataset were euploid rather than mosaic or aneuploid embryos [36, 37].

Variation and quality of data reporting to the SART-CORS database is another potential limitation. Of 51,273 cycles utilizing PGT-A for all embryos, only 10,778 cycles were included in the final analysis due to incomplete or inconsistent data. It is likely that several cycles initiated with intent for PGT may have been categorized as PGT cycles in the SART-CORS database, regardless of whether PGT was ultimately performed. For example, a small fraction of cycles specified as “PGT-A cycles” (1,205 cycles) resulted in fresh transfers and were excluded in the final analysis. The outcome of PGT-A testing (or whether PGT was ultimately performed) also remained ambiguous whether for embryo banking, oocyte banking, and/or frozen oocyte cycles, and thus these cycles were also excluded. Finally, only 2,100 of 3,809 cycles (55.1%) that resulted in aborted transfers due to no normal embryos following PGT-A, and only 8,778 of 16,401 cycles (53.5%) that resulted in a documented transfer attempt following PGT-A had sufficient linked index cycle information (such as AMH and number of embryos biopsied) that would allow for analysis. With nearly half of these cycles excluded due to incomplete data, data quality remains a concern that is partially mitigated by our stringent inclusion criteria.

**Fig. 2:**
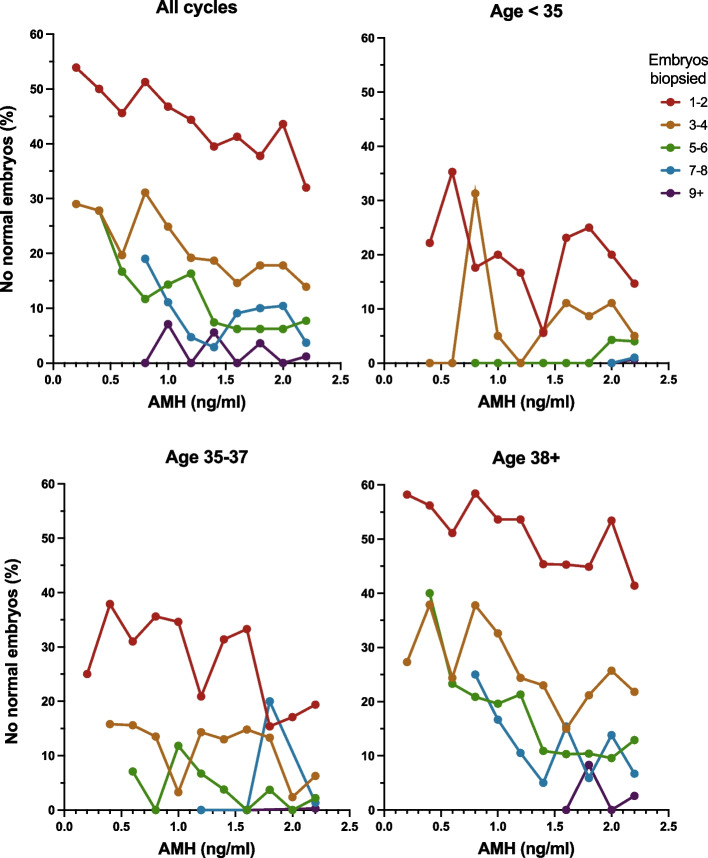
Risk of no normal embryos after PGT-A by AMH and Age

Our results support the recent findings of Jaswa et al. (2021) which showed convincing evidence of an association between DOR, as determined by Bologna criteria, and aneuploidy risk that was independent of age, though the predictive value of AMH alone was not reported [32]. Interestingly, our results extend this relationship between ovarian reserve and aneuploidy, showing that AMH appears to continue to have a predictive range even at values above the DOR range, i.e. values above 1.0 ng/ml (Fig. 2). This suggests that AMH as a quantitative marker has clinical utility beyond dichotomizing patients into groups with and without DOR [3, 9]. By directly assessing the predictive role of AMH, currently the most widely used marker of ovarian reserve, these data have greater clinical applicability to contemporary IVF practice, as well as to a broader population of patients (with and without a diagnosis of DOR).

Conversely, our results are not consistent with recent studies by Fouks et al. (2021) and Morin et al. (2018) [22, 33]. However, several relevant factors may explain these discrepant results. First, both studies utilized significantly smaller sample sizes; while our study captures 10,878 PGT-A cycles (for 10,020 unique patients), Fouks et al. and Morin et al. report data on 1,150 women (383 with DOR and 767 propensity-matched controls without DOR) and 2,103 women (345 with DOR and 1758 without DOR), respectively. Second, both studies exclude women from advanced age groups; Morin et al. included women with < 38 years old and Fouks et al. included women < 40 years old. The combination of smaller sample sizes and exclusion of patients at greatest risk of aneuploidy may reduce the power of each study to detect differences in the effect of AMH of aneuploidy risk. This is overall reflected in the lower incidence of aneuploidy in each sample; across the entire study sample, the aneuploidy rate per embryo biopsied was 29–30% for Morin et al. and 39–42% for Fouks et al. While a per-embryo aneuploidy rate could not be directly calculated in our sample, we estimate using numerical methods based on a 19.3% rate of no normal embryos and the distribution of embryos biopsied in our sample that the aneuploidy rate per embryo would be approximately 58.6%—considerably higher than that of both Morin et al. and Fouks et al. Importantly, both studies also model DOR as a dichotomous variable by either parsing the study population by percentile (AMH < 10^th^ percentile vs. 25-75^th^ percentile in Morin et al.), or by presence or absence of a physician-reported diagnosis of DOR (which the authors validated by a maximum AMH cut-off of 1.1 ng/ml). By modeling AMH as a continuous variable over the entire range represented in our study population, our data suggest persistent effects of AMH variation on aneuploidy risks even at values well above 1.1 ng/ml. Thus, the dichotomization of patients into groups with and without DOR based on low AMH cut-offs may cause significant effects of AMH variation above traditionally low cut-offs to evade statistical detection.

Comparing the relative predictive abilities of AMH and age for aneuploidy may have important clinical implications, especially for identifying patients who may benefit from PGT-A testing. In patients < 35 years, AMH was superior to age in predicting risk of no euploid embryos, with age being non-predictive. In patients ≥ 35 years, both age and AMH significantly predict risk of no euploid embryos, though AMH was the superior predictor only in patients 35–37 years, and age was by far the superior predictor in patients ≥ 38 years. Indeed, it is well-established that the relationship between maternal age and aneuploidy strengthens at advanced ages [23, 38].

Notably, our group has previously shown that, in non-PGT cycles from the SART-CORS database, AMH was an independent predictor of live birth in both fresh and frozen-thawed transfer cycles when controlling for multiple confounders, including age, BMI, race, day of transfer, and number of embryos transferred [39]. Taken together, this present study’s finding that AMH predicts aneuploidy but not live birth in PGT-A cycles supports the notion that the association between AMH and live birth in non-PGT cycles is not only due to a quantitative effect, but also a qualitative effect as reflected by the association between AMH and likelihood of embryo aneuploidy.

The finding that both age and AMH appear to be irrelevant predictors of live birth once a euploid embryo is identified is consistent with prior studies [22, 32, 33]. It also suggests that embryo aneuploidy (an outcome presumed to be eliminated by normal PGT-A testing) is a significant detrimental factor in live birth rates following transfer of untested embryos for patients of advanced age (> 35–38 years), and possibly for patients with diminished ovarian reserve as determined by AMH. This is consistent with evidence that patients of advanced age, particularly those considering single embryo transfers to reduce the risk of multiple pregnancies and its attendant risks, may stand to benefit the most from PGT-A [28, 40, 41]. Translated clinically, especially in the setting of single embryo transfers, patients ≥ 38 years may benefit from PGT-A testing, while patients 35–37 years may potentially benefit in the setting of diminished ovarian reserve. While further studies considering risk–benefit and cost-effectiveness analyses are needed to determine which patients are likely candidates for PGT-A, our findings suggest that AMH values may play an informative role, particularly in women 35–37 years old.

## Conclusions

While AMH is a predictor of live birth for non-PGT IVF cycles, it is unknown if this is due solely to quantitative factors or if qualitative factors contribute. Consistent with other recent studies investigating the relationship between diminished ovarian reserve, oocyte quality, and specifically embryo aneuploidy, this study suggests that AMH independently predicts likelihood of obtaining ≥ 1 euploid embryo for transfer in IVF PGT-A cycles after adjusting for age and number of embryos biopsied. However, neither age nor AMH are predictive of live birth per transfer. This analysis further suggests a predictive role for AMH on oocyte quality (aneuploidy risk), but not live birth per transfer once a euploid embryo is identified following PGT-A.

## Data Availability

This study uses data from the SART CORS database, available by request to SART CORS members via the SART research portal.

## Abbreviations

AICc: Corrected Akaike information criterion
AMH: Anti-Müllerian Hormone
aOR: Adjusted Odds Ratio
CI: Confidence Interval
DOR: Diminished Ovarian Reserve
FET: Frozen Embryo Transfer
FSH: Follicle Stimulating Hormone
ICSI: Intracytoplasmic Sperm Injection
IQR: Interquartile Range
IVF: In-vitro Fertilization
LR: Likelihood Ratio
PCOS: Polycystic Ovarian Syndrome
PGT-A: Preimplantation Genetic Testing for Aneuploid
SART-CORS: Society for Assisted Reproductive Technology Clinic Outcome Reporting System
SD: Standard Deviation
